# Perineural Steroid Injections in Cubital Tunnel Syndrome: A Systematic Review and Meta-Analysis

**DOI:** 10.7759/cureus.104271

**Published:** 2026-02-25

**Authors:** Amaar A Ali, Zubayr Ali, Ahmed Eid, Rahul Kakkar, Minahil Arshad

**Affiliations:** 1 Trauma and Orthopaedics, Morecambe Bay NHS Foundation Trust, Barrow, GBR; 2 Trauma and Orthopaedics, St George's University of London, London, GBR; 3 Trauma and Orthopaedics, University of Manchester, Manchester, GBR

**Keywords:** cubital tunnel, elbow joint, mononeuropathy, steroid injection, ulnar nerve

## Abstract

Cubital tunnel syndrome (CuTS) is a prevalent mononeuropathy, although the role of perineural steroid injections (PSI) remains uncertain. This meta-analysis and systematic review sought to compile and evaluate all available evidence regarding the potential benefits of PSI. The performance of PSI was assessed objectively using ultrasound (US) and nerve conduction studies NCS), and evaluated clinically through patient-reported questionnaires. This review followed the Preferred Reporting Items for Systematic Reviews and Meta-Analyses (PRISMA) guidelines. A literature search was performed across PubMed, Embase, and Medline databases. Common outcomes and control groups were categorised together. Meta-analysis was performed where possible, while narrative synthesis was used for studies with substantial heterogeneity. A risk of bias assessment was also undertaken.

The search yielded a total of 1751 papers. Nine studies met the inclusion criteria, encompassing 357 cases of CuTS. Clinically, patients who received PSI performed significantly better on clinical questionnaires and reported improved global symptom ratings. PSI was shown to be a useful adjunct compared with other treatment modalities, such as surgery and splinting. Steroid injections were also shown to be a reliable diagnostic tool in clinically uncertain cases of CuTS. No significant complication profile was identified with PSI. Meta-analysis of pooled results revealed no significant improvement in US findings or NCS.

PSI was found to alleviate symptoms and signs of CuTS across a range of clinical tools and questionnaires. It represents a safe therapeutic modality that could serve a valuable adjunctive and diagnostic function. Objective improvement on imaging or electromyographic studies was not observed. Additional longitudinal studies with larger sample sizes are required. There is also a need for further comparisons between different treatment options.

## Introduction and background

Cubital tunnel syndrome (CuTS) is the second-most frequent compressive neuropathy of the upper limb, caused by ulnar nerve compression at the elbow [1]. It typically manifests as numbness, tingling, and weakness in the ring and little fingers, and with prolonged or severe compression, may progress to intrinsic hand weakness and muscle wasting [2]. Mechanical factors such as repetitive elbow flexion, external pressure, anatomic narrowing of the cubital tunnel, nerve subluxation, and systemic conditions, including diabetes, all increase the likelihood of symptomatic entrapment [3].

Diagnosis is primarily clinical but is commonly supported by nerve conduction studies (NCS) and increasingly by high-resolution ultrasound (US) to identify the site of compression and quantify nerve cross-sectional area [4]. Initial management is conservative, including activity modification, night splints, and physiotherapy, but patients with persistent or progressive motor deficit are considered for surgical intervention. Multiple operative techniques exist, such as in-situ decompression, anterior transposition, and epicondylectomy [5]. Although most patients improve after surgery, a minority experience persistent symptoms, early postoperative worsening, or require revision surgery; these issues are variably related to delayed neural recovery, perineural inflammation, or technical and stability-related complications [6]. 

In other peripheral nerve compression disorders, most notably carpal tunnel syndrome (CTS), perineural steroid injections (PSI) have long shown significant clinical benefit, supporting their potential utility in cubital tunnel disease. Corticosteroids reduce intraneural oedema, suppress inflammatory cytokine activity, and improve median nerve gliding, resulting in short-term improvements in nerve conduction and symptom relief [7,8]. Randomised trials and meta-analyses consistently show that steroid injections in CTS provide superior symptom improvement for up to several months, with meaningful reductions in pain, nocturnal paraesthesia, and functional limitation [9,10]. US-guided administration has further enhanced efficacy by ensuring optimal perineural distribution and promoting faster early recovery [11]. Although effects may diminish over time, evidence indicates that steroid therapy delays the need for surgical release in many patients and offers a safe, low-risk period for effective symptom management [12,13].

Despite the potentially promising effects of PSI in other compressive neuropathies, evidence for its use in CuTS remains limited and fragmented. Existing studies are restricted by small sample sizes and methodological variability, including differences in injection technique, corticosteroid formulation, and use of image guidance. This contributes to inconsistent reporting of both clinical and objective outcomes [14]. Given that many people with CuTS wish to avoid surgery and that postoperative improvement can be gradual with occasional persistent symptoms, there is a need to determine whether PSI can provide meaningful symptom relief or delay the need for invasive intervention [15]. This systematic review and meta-analysis synthesises current clinical, US, and NCS data to evaluate the effect of steroid injections in CuTS and to assess their safety within the existing literature.

## Review

Methods

This systematic review and meta-analysis adhered to the Preferred Reporting Items for Systematic Reviews and Meta-Analyses (PRISMA) guidelines [16]. Two reviewers (AA and ZA) developed a broad search strategy to maximise the yield of papers. The full search term is provided in the Appendices. Three databases were searched: PubMed, Medline, and Embase. The search included all studies published up to December 2025. Grey literature was excluded to ensure high-quality, peer-reviewed evidence. All results were imported into the systematic review management software, ‘Covidence,’ which automatically removed duplicates. Before screening, clear inclusion and exclusion criteria were established by the reviewers (Table 1). In ‘Covidence,’ all papers underwent an initial round of title and abstract screening. All imported papers were screened by two reviewers (AA and ZA). If disagreement arose regarding the inclusion or exclusion of a study, the decision to proceed to full-text screening was referred to a third reviewer (MA).

**Table 1:** Inclusion and exclusion criteria EMG: electromyography

	Inclusion criteria	Exclusion criteria
Population	Patients with clinical or objective evidence of cubital tunnel syndrome	Other mononeuropathies, ulnar nerve compression at sites other than the elbow
Intervention	Perineural steroid injections at the elbow (inc. adjunctive role, coupled with other treatments)	Oral steroids, injections elsewhere
Comparison	-	-
Outcome	Clinical assessment/EMG data/ultrasound findings/complications	Anything else
Study design	Academic literature and the English language	Grey literature, abstracts, reviews, case reports, animal studies, other languages

After completing ‘Title and Abstract’ screening, each paper underwent full-text screening, with the same inclusion and exclusion criteria applied as before. Every paper was reviewed by two reviewers, and if disagreement arose regarding its relevance, the decision was referred to a third reviewer (MA). All selected papers had their references checked for additional studies that could potentially meet the inclusion/exclusion criteria. The included studies then underwent data extraction. Using ‘Google Sheets,’ a table was designed collaboratively by the three reviewers (AA, ZA, and MA) and subsequently populated with the required information.

A risk-of-bias assessment was conducted for each paper. For randomised controlled trials, the Jadad scale [17] was used, while for observational studies, the Modified Newcastle-Ottawa scale [18] was applied. Bias was not used as a criterion for excluding papers from this systematic review and meta-analysis, in order to maximise the breadth of available evidence. Following data extraction, two reviewers (AA and ZA) conducted thematic analysis on the included studies using Braun and Clarke’s six steps [19]. Where there was sufficient homogeneity in comparators, methods, and outcomes, meta-analysis was performed using the statistical software ‘JASP’. A random-effects model was applied, with the estimator set as restricted maximum likelihood. Effect sizes were calculated using standardised mean differences. Papers unsuitable for meta-analysis, due to heterogeneous outcomes or comparators, were included in the narrative synthesis.

Results

**Figure 1:**
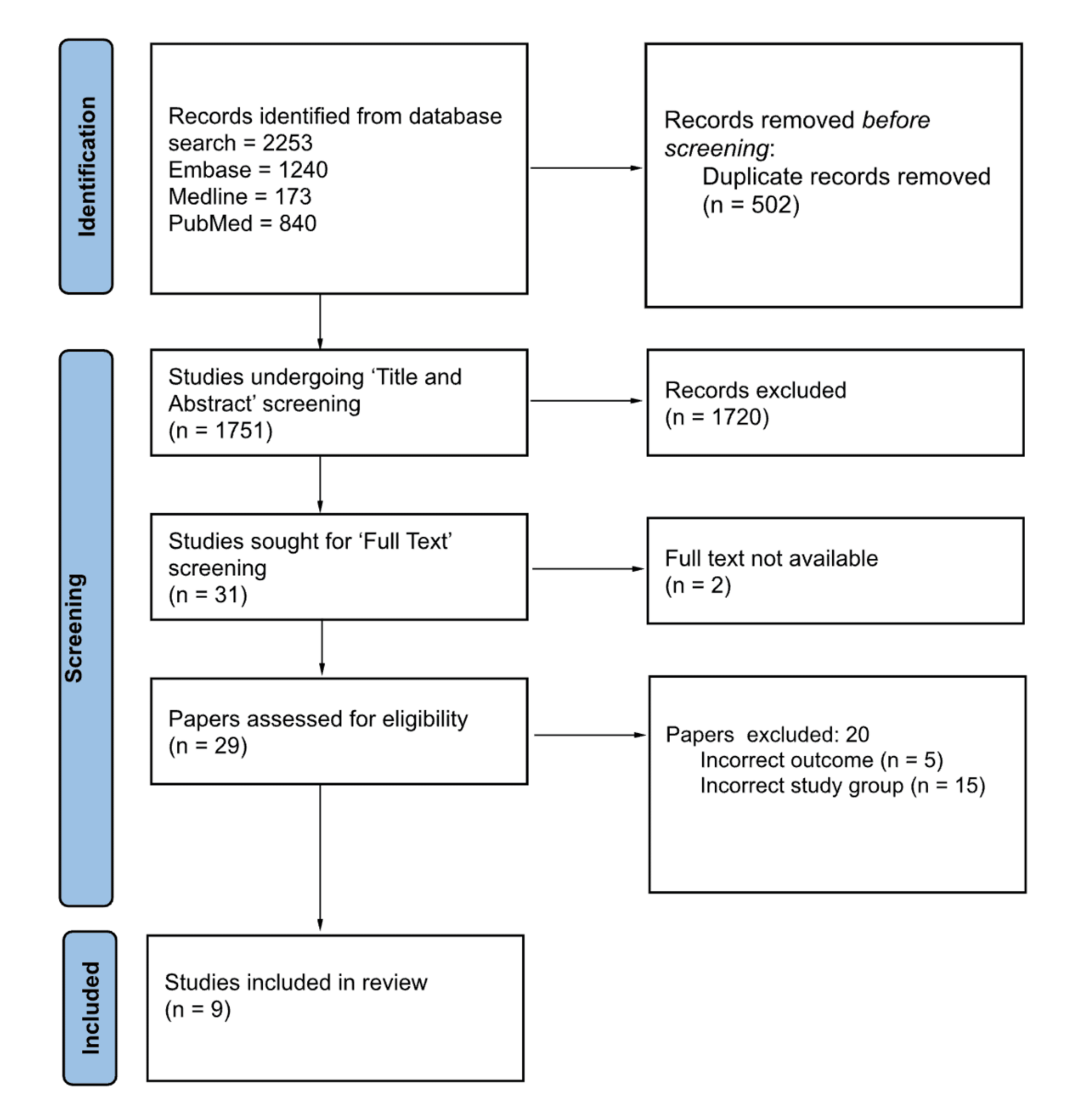
PRISMA diagram depicting the selection of studies PRISMA: Preferred Reporting Items for Systematic Reviews and Meta-Analyses

The search of three databases yielded 2,253 records, from which 502 duplicate records were removed. A total of 1,751 studies then underwent title and abstract screening, leaving 31 studies for full-text screening. Following full-text screening, nine studies advanced to data extraction. No additional relevant papers were identified through manual screening of the reference lists of the included studies. This process is summarised in the PRISMA flow diagram shown in Figure 1.

Table 2 presents the general characteristics of the included studies. Of the nine studies in this systematic review and meta-analysis, four were randomised controlled trials [20-23], and five were observational studies [24-28]. The median year of publication was 2015. The total sample size was 357 ulnar nerves, including 175 (49%) in males and 188 (51%) in females. The average age of participants was 50.7 years. The average maximum follow-up period was 3.9 months. Four studies administered triamcinolone acetonide [20,21,22,25], three studies used methylprednisolone acetate [23,24,28], one study used betamethasone [26], and one study used dexamethasone [27]. A variety of comparators were employed alongside steroid injection, including platelet-rich plasma [20], dextrose [21], splinting [22], anterior transposition [27], and placebo [23]. In studies without a comparator group, outcomes were assessed relative to baseline measurements within the intervention group.

**Table 2:** Summary of individual studies

First author	Year	Study type	Population size	Study group	Comparator	Outcomes
El Naggar et al. [20]	2023	Randomised controlled trial	60	Triamcinolone acetonide	Platelet-rich plasma	Nerve conduction study, ultrasound, questionnaire
Chen et al. [21]	2020	Randomised controlled trial	33	Triamcinolone acetonide	Dextrose injection	Nerve conduction study, ultrasound, questionnaire
Hong et al. [22]	1996	Randomised controlled trial	12	Triamcinolone acetonide (+splinting)	Splinting alone	Nerve conduction study and questionnaire
vanVeen et al. [23]	2015	Randomised controlled trial	49	Methylprednisolone acetate	Placebo	Nerve conduction study, ultrasound, questionnaire
Albas et al. [24]	2012	Observational	9	Methylprednisolone acetate	Nil	Nerve conduction study, ultrasound, questionnaire
Choi et al. [25]	2015	Observational	10	Triamcinolone acetonide	Nil	Nerve conduction study, ultrasound, questionnaire
Gronbeck et al. [26]	2021	Observational	63	Betamethasone	Nil	Questionnaire, need for surgery
Hu et al. [27]	2025	Observational	114	Dexamethasone (+anterior transposition)	Anterior transposition alone	Nerve conduction study and questionnaire
Rampen et al. [28]	2011	Observational	7	Methylprednisolone acetate	Nil	Ultrasound and questionnaire

Meta-Analysis

Table 3 presents the results of the meta-analysis. Following data extraction, three outcomes were identified as suitable for meta-analysis: nerve cross-sectional area (CSA) on US (mm²), motor nerve conduction velocity (MNCV) (m/s), and compound motor action potential (CMAP) (mV) on NCS. Four studies assessed CSA [20,21,24,28], six assessed MNCV [20,21,22,24,25,27], and four assessed CMAP [20,22,25,27]. The meta-analysis was limited by insufficient data for shared comparators, such as alternative treatment modalities, including steroid vs splinting. Consequently, the analysis was restricted to paired comparisons of pre- and post-treatment outcomes following steroid injection, without a true control group. Sensitivity analysis and subgroup analysis were not performed due to the small number of included studies, as undertaking these analyses would have rendered the meta-analysis infeasible and statistically underpowered. 

**Table 3:** Overview of meta-analysis

	Nerve cross-sectional area	Motor nerve conduction velocity	Compound motor action potential
Number of studies	4	6	4
Intervention vs comparator	Steroid vs baseline	Steroid vs baseline	Steroid vs baseline
Pooled population size	62	132	107
Effect size (95% CI)	0.96 (-0.39, 2.31)	-0.49 (-2.58, 1.59)	2.46 (-4.36, 9.27)
Combined effect size p-value	0.109	0.571	0.335
I2	75.0 (95% CI 15.0-98.3)	96.3 (95% CI 90.5-99.4)	99.1 (95% CI 97.1-99.9)

**Figure 2:**
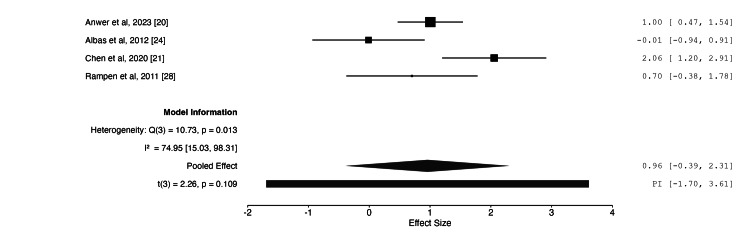
Forest plot of changes in ulnar nerve cross-sectional area following steroid injection Effect size with 95% CI shown on the right. Negative effect size = improvement

**Figure 3:**
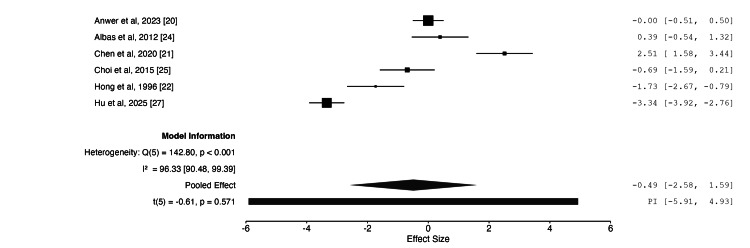
Forest plot of changes in ulnar nerve motor nerve conduction velocity following steroid injection Effect size with 95% CI shown on the right. Positive effect size = improvement

**Figure 4:**
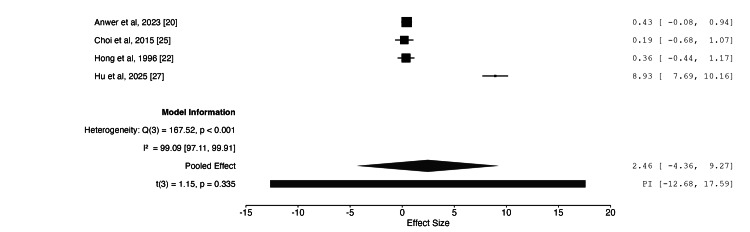
Forest plot of changes in ulnar nerve compound motor action potential following steroid injection Effect size with 95% CI shown on the right. Positive effect size = improvement

The meta-analysis demonstrated no statistically significant effect on CSA following steroid injection, with a p-value of 0.109 and an effect size of 0.96 (95% CI: -0.39, 2.31). For MNCV, the effect size was -0.49 (95% CI: -2.58, 1.59), which was not statistically significant (p = 0.571). For CMAP, the effect size was 2.46 (95% CI: -4.36, 9.27), again indicating no statistically significant difference (p = 0.335). Moreover, substantial heterogeneity was observed across all three outcomes, with I² values exceeding 75% in each case. This may be attributable to the small number of included studies, many of which had limited sample sizes. Figures 2-4 illustrate the forest plots for each of the three outcomes included in the meta-analysis.

Global Assessment of Symptoms

Four papers [23,24,26,28] required patients to provide a self-reported global assessment of symptoms following PSI. When asked whether their symptoms had worsened, remained unchanged, or improved, five of nine patients in Albas et al.’s [24] study reported improvement at three months compared with baseline, while one patient reported worsening symptoms. Rampen et al. [28] employed a similar 3-point symptom change scale. Four of seven patients demonstrated improvement at six weeks of follow-up, and one patient experienced worsening symptoms. Gronbeck et al. [26] used a global rating scale with four categories: complete improvement, partial improvement, no change, and worsening. Thirty-eight of 66 patients reported either partial (22) or complete (16) improvement. However, 26 of these patients experienced symptom recurrence within three months of follow-up. Three patients reported worsening symptoms. Van Veen et al. [23] used a 6-point scale to evaluate symptom change. Nine of 30 patients receiving PSI achieved a favourable outcome, defined as complete, partial, or slight improvement, whereas the remaining patients were classified as having unfavourable outcomes, including no improvement, some worsening, or deterioration. One patient reported a slight worsening of symptoms. Compared with placebo, there was no statistically significant difference in the proportion of patients achieving favourable outcomes following PSI.

Semi-quantitative Measures

An array of clinical tools/questionnaires to semi-objectively rate the severity of CuTS was adopted by different studies. The Michigan Hand Questionnaire was utilised in one study. Anwer et al. [20] reported a significant improvement at one and three months compared with baseline in the group receiving steroid injections. When comparing the steroid group with the comparator group receiving platelet-rich plasma, a statistically significant difference was observed only at one month. Chen et al. [21] used the Disabilities of the Arm, Shoulder, and Hand (DASH) questionnaire. When comparing patients treated with perineural steroid injections to those receiving dextrose injections, no significant difference in DASH scores was identified at any follow-up time point. However, at one and three months following PSI, patients in the steroid group demonstrated significant improvement relative to baseline, but no significant result was found beyond this.

The SQUINE questionnaire (self-administered questionnaire of ulnar neuropathy at the elbow) and the McGowan Classification were deployed by Choi et al. [25]. No significant difference in the score for either clinical tool was found for patients receiving PSI compared to their baseline at any follow-up time point. Hu et al. [27] compared patients receiving PSI with another cohort receiving adjunctive steroid injections at the time of anterior nerve transposition. The PRUNE (Patient-Reported Ulnar Nerve Evaluation) score was found to be significantly greater in the group undergoing surgery with adjunctive steroid treatment at one month and six months post-op. When using the Modified Bishop score, similar significant outcomes were noted at both follow-up time points. 

The Visual Analogue Scale (VAS) was used in three studies [21,25,27]. Hu et al. [27] found a significant difference in the VAS score for patients receiving surgery and PSI compared to another group receiving PSI alone at the one-month follow-up, but not at six months. Chen et al. [21] found no significant difference when using VAS to compare patients receiving PSI with dextrose injections at any follow-up period. However, within the steroid group, the VAS score significantly improved compared to the group’s baseline up to six months after treatment. Similar results were found by Choi et al. [25]. When using the VAS score, patients receiving PSI had significant improvements in symptoms compared to their baseline at one week and four weeks of follow-up. Hong et al. [22] designed their own clinical questionnaire to evaluate patients' signs and symptoms. They compared patients receiving an elbow splint and PSI with those receiving an elbow splint alone. At one and six months, there was significant improvement in symptoms, but not signs, for the splint plus PSI group compared to their baseline. No significant difference was found between groups at any time point.

Surgery

One study [26] investigated whether PSI could identify suitable candidates for surgery. Gronbeck et al. [26] found 13 patients who had partial improvement with steroid therapy, whose symptoms recurred soon after injection. These 13 patients then underwent surgery, and all showed complete resolution of symptoms, potentially highlighting a diagnostic role for PSI. Hu et al. [27] evaluated whether PSI could play an adjunctive role in surgical patients. They compared patients undergoing anterior transposition with another group undergoing anterior transposition plus a single PSI administered intraoperatively. The steroid plus surgery group showed significantly better PRUNE and Modified Bishop scale scores at four weeks and six months. Nerve conduction studies also showed significantly improved MNCV and CMAP at six months.

Ultrasound

Vanveen et al. [23] was the only study to find a significant difference in CSA between PSI and a comparator, a placebo. Anwer et al. [20] and Chen et al. [21] found no significant change in CSA for PSI compared to platelet-rich plasma injections and dextrose injections, respectively. As reported previously, the meta-analysis showed no significant improvement for PSI in reducing CSA compared to pre-injection values. 

Motor Nerve Conduction Velocity 

When Hu et al. [27] compared patients undergoing anterior transposition of the ulnar nerve with another cohort undergoing the same procedure plus an intraoperative steroid injection, a significant difference in MNCV was found at six months follow-up. Anwer et al. [20], Chen et al. [21], and VanVeen et al. [23] found no significant difference, even when compared with platelet-rich plasma, dextrose injections, and placebo, respectively. Hong et al. [22] found no statistically significant difference in MNCV when comparing patients wearing a splint with those wearing a splint plus receiving PSI. As highlighted by the meta-analysis, no significant difference in MNCV was found when comparing patients post-steroid injection with their baseline readings.

*Compound Motor Action Potential* 

When Hu et al. [27] compared patients undergoing anterior transposition of the ulnar nerve with those receiving the same procedure plus an intraoperative PSI, a significant improvement in CMAP was observed at six months of follow-up. Anwer et al. [20] reported no statistically significant change in CMAP when comparing patients receiving PSI with those receiving platelet-rich plasma injections. Similarly, Hong et al. [22] found no significant difference in CMAP between patients treated with a splint alone and those treated with a splint plus PSI. Consistent with the findings of the meta-analysis, no significant change in CMAP was detected when comparing post-steroid injection values with baseline measurements.

Risk-of-Bias

The Jadad scale [17] was applied to evaluate the risk of bias in the included randomised controlled trials. The assessment results are presented in Table 4. For observational studies, risk of bias was assessed using the modified Newcastle-Ottawa scale [18], with the results shown in Table 5. 

**Table 4:** Risk-of-bias assessment for randomised control trials using the Jadad score

	El Naggar [20]	Chen [21]	Hong [22]	vanVeen [23]
Randomisation /2	2	2	1	2
Blinding /2	2	2	0	2
Withdrawal and dropouts /1	0	1	0	1
Total score /5	4	5	1	5

**Table 5:** Risk-of-bias assessment for observational studies using the modified Newcastle-Ottawa scale

	Albas [24]	Choi [21]	Gronbeck [22]	Hu [27]	Rampen [28]
Selection	d, c, a*, a*	d, c, a*, a*	d, c, a*, a*	d, a*, a*, a*	d,c, a*, b
Comparability	Nil	Nil	Nil	Nil	Nil
Outcome	c, a*, a*	c, a*, a*	c, a*, a*	c, a*, b*	c, a*, a*
Total /9*	4*	4*	4*	5*	3*

Discussion

Our meta-analysis indicates that PSI does not appear to produce meaningful improvements in objective surrogate measures, such as US and NCS, for CuTS. It is important to note the absence of a control group in this analysis. Due to limited data, comparisons were restricted to pre- and post-steroid injection outcomes, which limits the ability to draw definitive conclusions about treatment efficacy. For CTS, the available evidence indicates a significant effect of steroids in reducing nerve CSA [29-31]. In CTS, a recent Cochrane review found that NCS is likely to improve following PSI [32]. The lack of comparable positive findings in CuTS may be explained by heterogeneity in follow-up intervals across studies, short follow-up durations, and small sample sizes. Larger, longer-term randomised controlled trials are required to more reliably evaluate objective outcomes such as US and NCS in CuTS.

On subjective symptom reporting, there is more encouraging evidence for the potential benefits of PSI in CuTS. This is supported by a recent meta-analysis, which showed that 59% of patients with CuTS receiving steroid injections experienced symptom improvement [33]. Only one study [26] conducted two rounds of a ‘global assessment’ and found that most patients who initially improved experienced a recurrence of symptoms at the second follow-up. This highlights an important gap in the current evidence. There is a general lack of extended follow-up in most studies, making it difficult to assess the trajectory of symptomatic improvement in patients receiving steroid injections.

Across a variety of clinical questionnaires and assessment tools, patients demonstrated significant improvement following steroid injection compared with their baseline scores. These instruments may provide more robust evidence of clinical benefit than a simple global rating alone, as they capture a broader range of information, including clinical examination findings and physical appearance. Additionally, the use of statistical tests allows for formal validation of differences in scores. It should be noted that although patients showed improved scores relative to baseline after steroid injection, comparisons with alternative treatment modalities revealed weaker evidence for a statistically significant advantage.

Two studies [22,27] investigated combining therapies to explore whether steroid injections can serve an adjunctive role. Hu et al. [27] administered a PSI intraoperatively during anterior transposition surgery, while Hong et al. [22] combined PSI with wearing a splint. Both studies showed positive outcomes in improving clinical signs and symptoms compared to monotherapy alone. This information may be useful in demonstrating compounded effects when treatment strategies are combined. More evidence is needed, but combining non-invasive treatments could offer greater benefit for patients not suitable for surgery. PSI could also act as a screening tool before surgery in equivocal cases of CuTS. Gronbeck et al. [26] recommended surgery for patients who had only partial improvement following steroid injections, and all showed significant symptomatic improvement. This suggests that when a diagnosis is uncertain, a steroid injection may provide clarity before undertaking invasive and irreversible interventions.

One key limitation in the evidence is the lack of head-to-head comparisons with existing treatment modalities. Conservative measures are generally tried before surgery. How PSI compares to splinting, NSAIDs, and activity modification is unclear. Although the depth of evidence for other non-invasive measures is generally better established, there is no clear hierarchy of which strategies are superior [33]. Practically speaking, steroid injections may be less desirable compared to other non-invasive interventions. Ultrasound guidance requires specialised expertise, which may limit accessibility. From a patient perspective, an injection is more invasive than a splint and may cause pain and bleeding. Ideally, qualitative analyses of operator and patient preferences are needed. It is important to note that no study reported a significant complication profile for PSI.

One key drawback of the evidence is the limited duration and frequency of follow-up. Current evidence for CTS shows clinical improvement in symptoms up to eight weeks post-injection [3,34,35]. Studies with longer follow-up periods and more frequent assessment intervals would allow for a more accurate depiction of the typical progress patients can be expected to make with PSI in CuTS.

## Conclusions

The evidence from this review indicates that PSI can lead to improvements in the signs and symptoms of CuTS, although corresponding improvements in objective measures such as US and NCS were not observed. PSI may have potential value as a diagnostic tool and as an adjunct to other treatment modalities. Further research is required to provide direct comparisons with alternative therapies and to generate longitudinal data on the duration of symptomatic improvements, which would help inform optimal scheduling of injections.

## Appendices

Appendix 1: search terms

(ulnar nerve OR cubital) AND (steroid OR corticosteroid OR dexamethasone OR prednisolone OR prednisone OR methylprednisone OR methylprednisolone OR hydrocortisone OR triamcinolone).

## References

[1] Burahee AS, Sanders AD, Shirley C, Power DM (2021). Cubital tunnel syndrome. EFORT Open Rev.

[2] Graf A, Ahmed AS, Roundy R, Gottschalk MB, Dempsey A (2023). Modern treatment of cubital tunnel syndrome: evidence and controversy. J Hand Surg Glob Online.

[3] Anderson D, Woods B, Abubakar T (2022). A comprehensive review of cubital tunnel syndrome. Orthop Rev (Pavia).

[4] Prasetyo M, Rahardja RR, Yanuar A, Prihartono J, Setiawan SI (2021). Ultrasonography evaluation of the normal ulnar nerve in adult: comparison of the cross-sectional area at elbow extension and flexion. Eur J Radiol Open.

[5] Wade RG, Griffiths TT, Flather R, Burr NE, Teo M, Bourke G (2020). Safety and outcomes of different surgical techniques for cubital tunnel decompression: a systematic review and network meta-analysis. JAMA Netw Open.

[6] Hewson DW, Kurien T, Hardman JG (2023). Postoperative ulnar neuropathy: a systematic review of evidence with narrative synthesis. Br J Anaesth.

[7] Urits I, Gress K, Charipova K, Orhurhu V, Kaye AD, Viswanath O (2019). Recent advances in the understanding and management of carpal tunnel syndrome: a comprehensive review. Curr Pain Headache Rep.

[8] Joshi A, Patel K, Mohamed A (2022). Carpal tunnel syndrome: pathophysiology and comprehensive guidelines for clinical evaluation and treatment. Cureus.

[9] Marshall S, Tardif G, Ashworth N (2007). Local corticosteroid injection for carpal tunnel syndrome. Cochrane Database Syst Rev.

[10] Ashworth NL, Bland JDP, Chapman KM (2023). Local corticosteroid injection versus placebo for carpal tunnel syndrome. Cochrane Database Syst Rev.

[11] Yang FA, Shih YC, Hong JP, Wu CW, Liao CD, Chen HC (2021). Ultrasound-guided corticosteroid injection for patients with carpal tunnel syndrome: a systematic review and meta-analysis of randomized controlled trials. Sci Rep.

[12] Babaei-Ghazani A, Roomizadeh P, Forogh B (2018). Ultrasound-guided versus landmark-guided local corticosteroid injection for carpal tunnel syndrome: a systematic review and meta-analysis of randomized controlled trials. Arch Phys Med Rehabil.

[13] Wolny T, Linek P (2019). Long-term patient observation after conservative treatment of carpal tunnel syndrome: a summary of two randomised controlled trials. PeerJ.

[14] Caliandro P, La Torre G, Padua R, Giannini F, Padua L (2016). Treatment for ulnar neuropathy at the elbow. Cochrane Database Syst Rev.

[15] Tong J, Dong Z, Xu B, Zhang C, Gu Y (2018). Predictors of surgical outcomes for severe cubital tunnel syndrome: a review of 146 patients. Acta Neurochir (Wien).

[16] (2026). PRISMA statement. https://www.prisma-statement.org/.

[17] Jadad AR, Moore RA, Carroll D, Jenkinson C, Reynolds DJ, Gavaghan DJ, McQuay HJ (1996). Assessing the quality of reports of randomized clinical trials: is blinding necessary?. Control Clin Trials.

[18] Wells GA, Shea B, O’Connell D (2026). The Newcastle-Ottawa Scale (NOS) for assessing the quality of nonrandomised studies in meta-analyses. 07 Jan.

[19] Braun V, Clarke V (2006). Using thematic analysis in psychology. Qual Res Psychol.

[20] El Naggar HA, Sergany ME, Alashkar DS, Waseem D (2023). Efficacy of ultrasound-guided deep perineural platelet rich plasma versus corticosteroid injection in patients with ulnar neuropathy at the elbow: a comparative randomized trial. Egypt J Hosp Med.

[21] Chen LC, Ho TY, Shen YP, Su YC, Li TY, Tsai CK, Wu YT (2020). Perineural dextrose and corticosteroid injections for ulnar neuropathy at the elbow: a randomized double-blind trial. Arch Phys Med Rehabil.

[22] Hong CZ, Long HA, Kanakamedala RV, Chang YM, Yates L (1996). Splinting and local steroid injection for the treatment of ulnar neuropathy at the elbow: clinical and electrophysiological evaluation. Arch Phys Med Rehabil.

[23] vanVeen KE, Alblas KC, Alons IM (2015). Corticosteroid injection in patients with ulnar neuropathy at the elbow: a randomized, double-blind, placebo-controlled trial. Muscle Nerve.

[24] Alblas CL, van Kasteel V, Jellema K (2012). Injection with corticosteroids (ultrasound guided) in patients with an ulnar neuropathy at the elbow, feasibility study. Eur J Neurol.

[25] Choi CK, Lee HS, Kwon JY, Lee WJ (2015). Clinical implications of real-time visualized ultrasound-guided injection for the treatment of ulnar neuropathy at the elbow: a pilot study. Ann Rehabil Med.

[26] Gronbeck C, Wolf J, Rodner CM (2021). Ultrasound-guided cubital tunnel injection: techniques and review of the current evidence. Tech Orthop.

[27] Hu T, Bian Y, Zhou T (2025). Improving short‑term outcomes of cubital tunnel syndrome decompression with intraoperative dexamethasone. World Neurosurg.

[28] Rampen AJ, Wirtz PW, Tavy DL (2011). Ultrasound-guided steroid injection to treat mild ulnar neuropathy at the elbow. Muscle Nerve.

[29] Lee YS, Choi E (2017). Ultrasonographic changes after steroid injection in carpal tunnel syndrome. Skeletal Radiol.

[30] Cartwright MS, White DL, Demar S (2011). Median nerve changes following steroid injection for carpal tunnel syndrome. Muscle Nerve.

[31] Jeong JS, Yoon JS, Kim SJ, Park BK, Won SJ, Cho JM, Byun CW (2011). Usefulness of ultrasonography to predict response to injection therapy in carpal tunnel syndrome. Ann Rehabil Med.

[32] Ashworth NL, Bland JD, Chapman KM, Tardif G, Albarqouni L, Nagendran A (2023). Local corticosteroid injection versus placebo for carpal tunnel syndrome. Cochrane Database Syst Rev.

[33] Natroshvili T, van de Warenburg MS, Heine EP, Slater NJ, Walbeehm ET, Bartels RH (2023). Conservative treatment of ulnar nerve compression at the elbow: a systematic review and meta-analysis. Arch Plast Surg.

[34] Andrews K, Rowland A, Pranjal A, Ebraheim N (2018). Cubital tunnel syndrome: anatomy, clinical presentation, and management. J Orthop.

[35] Nakashian MN, Ireland D, Kane PM (2020). Cubital tunnel syndrome: current concepts. Curr Rev Musculoskelet Med.

